# Plasma levels of soluble TREM2 and neurofilament light chain in *TREM2* rare variant carriers

**DOI:** 10.1186/s13195-019-0545-5

**Published:** 2019-11-28

**Authors:** Nicholas J. Ashton, Marc Suárez-Calvet, Amanda Heslegrave, Abdul Hye, Cristina Razquin, Pau Pastor, Raquel Sanchez-Valle, José L. Molinuevo, Pieter Jelle Visser, Kaj Blennow, Angela K. Hodges, Henrik Zetterberg

**Affiliations:** 1 Department of Psychiatry and Neurochemistry, Institute of Neuroscience & Physiology, The Sahlgrenska Academy at the University of Gothenburg, Mölndal, Sweden; 2 Wallenberg Centre for Molecular and Translational Medicine, University of Gothenburg, Gothenburg, Sweden; 3 Institute of Psychiatry, Psychology & Neuroscience, King’s College London, London, UK; 4 NIHR Biomedical Research Centre for Mental Health & Biomedical Research Unit for Dementia at South London & Maudsley NHS Foundation, London, UK; 5 Barcelonaβeta Brain Research Center (BBRC), Pasqual Maragall Foundation, Barcelona, Catalonia Spain; 6 Department of Neurology, Hospital del Mar, Barcelona, Catalonia Spain; 7 Department of Neurodegenerative Disease, UCL Institute of Neurology, London, UK; 8 UK Dementia Research Institute at UCL, London, UK; 9 Department of Preventive Medicine and Public Health, University of Navarra, Pamplona, Spain; 10 Instituto de Investigación Sanitaria de Navarra, Pamplona, Spain; 11 CIBER and Fisiopatología de la Obesidad y Nutrición (CIBERobn), Instituto de Salud Carlos III, Madrid, Spain; 12 Fundació Docència i Recerca Mútua Terrassa, University Hospital Mútua de Terrassa, Terrassa, 08221 Barcelona, Spain; 13 Movement Disorders Unit, Department of Neurology, University Hospital Mútua de Terrassa, Terrassa, 08222 Barcelona, Spain; 14 Alzheimer’s Disease and Other Cognitive Disorders Unit, Neurology Service, Hospital Clínic, Barcelona, Spain; 15 August Pi i Sunyer Biomedical Research Institute (IDIBAPS), Barcelona, Spain; 16 Alzheimer Center Amsterdam, Department of Neurology, Amsterdam Neuroscience, Vrije Universiteit Amsterdam, Amsterdam UMC, Amsterdam, the Netherlands; 17 Alzheimer Center Limburg, School for Mental Health and Neuroscience, Maastricht University, Maastricht, the Netherlands; 18 Clinical Neurochemistry Laboratory, Sahlgrenska University Hospital, Mölndal, Sweden

**Keywords:** Alzheimer’s disease, sTREM2, Blood, Biomarkers, Neurofilament light chain

## Abstract

**Background:**

Results from recent clinical studies suggest that cerebrospinal fluid (CSF) biomarkers that are indicative of Alzheimer’s disease (AD) can be replicated in blood, e.g. amyloid-beta peptides (Aβ_42_ and Aβ_40_) and neurofilament light chain (NFL). Such data proposes that blood is a rich source of potential biomarkers reflecting central nervous system pathophysiology and should be fully explored for biomarkers that show promise in CSF. Recently, soluble fragments of the triggering receptor expressed on myeloid cells 2 (sTREM2) protein in CSF have been reported to be increased in prodromal AD and also in individuals with *TREM2* rare genetic variants that increase the likelihood of developing dementia.

**Methods:**

In this study, we measured the levels of plasma sTREM2 and plasma NFL using the MesoScale Discovery and single molecule array platforms, respectively, in 48 confirmed *TREM2* rare variant carriers and 49 non-carriers.

**Results:**

Our results indicate that there are no changes in plasma sTREM2 and NFL concentrations between *TREM2* rare variant carriers and non-carriers. Furthermore, plasma sTREM2 is not different between healthy controls, mild cognitive impairment (MCI) or AD.

**Conclusion:**

Concentrations of plasma sTREM2 do not mimic the recent changes found in CSF sTREM2.

## Introduction

There has been considerable progress in the search for blood-based biomarkers able to capture the clinical course and underlying pathophysiology of Alzheimer’s disease (AD), for review see [1]. Reduced plasma Aβ_42_/Aβ_40_ ratio [2–4] and increased neurofilament light chain (NFL) [5–7] are becoming consistently reported in AD, and encouragingly, these findings mimic the more established observations seen in cerebrospinal fluid (CSF) [8]. NFL is not a specific biomarker for AD [9]. Increases are observed in a number of neurodegenerative disorders [10–12], owing to its global reflection of axonal injury or degeneration. Yet, translating other co-pathology markers of neurodegeneration from CSF to blood has been less successful. For example, plasma total tau (T-tau) increases in AD [13] but it seems to have limited clinical utility, and phosphorylated form of tau (P-tau) has proven difficult to establish as a reliable measure in blood despite recent promise [14]. Likewise, TDP-43 [15], alpha-synuclein [16] and the post-synaptic dendritic biomarker, neurogranin [17], all have substantial and specific peripheral expression but levels appear to be unrelated to changes in the central nervous system.

The triggering receptor expressed on myeloid cells 2 (TREM2) is an innate immune receptor that guides essential functions of microglia. Rare variants in *TREM2* strongly increase the likelihood of developing AD, frontotemporal dementia (FTD), Parkinson’s disease (PD) and amyotrophic lateral sclerosis (ALS) [18–23]. TREM2 is a type 1 transmembrane protein, and its ectodomain is shed at the plasma membrane by ADAM family proteases C-terminal at histidine 157 position [24–27]. The resulting soluble fragment (sTREM2) is released into the extracellular space and can be found in CSF and plasma [28, 29]. Recently, the concentrations of CSF sTREM2 have been shown to be increased in early symptomatic stages of sporadic [30–34] and autosomal dominant AD patients [35]. Interestingly, Aβ pathology and tau-related neurodegeneration may impact levels of CSF sTREM2 differently [33]. Moreover, it has been shown that the concentrations of CSF sTREM2 vary between different disease-associated *TREM2* genetic variant carriers [32, 33].

Unlike CSF sTREM2, levels of sTREM2 in blood have been poorly investigated. In this study, we investigate plasma concentrations of sTREM2 in patients with AD and mild cognitive impairment (MCI) compared with aged-matched healthy controls. Furthermore, in a novel approach, we also report on blood concentrations of sTREM2 and NFL in *TREM2* rare variant carriers.

## Methods

### Participants

Samples from a total of 97 participants were used for these analyses (Table 1). The majority of samples (*n* = 82) were from the longitudinal AD cohorts managed at King’s College London (KCL; AddNeuroMed [36], Kings Health Partners-Dementia Case Register (KHP-DCR) a UK clinic and population based study [37] and the MRC AD Biomarker study [38]). Further samples were obtained from DEGESCO (*n* = 11, Dementia Genetics Spanish Consortium [39, 40]) and EDAR (*n* = 4, beta amyloid oligomers in the early diagnosis of AD and as marker for treatment response [41]). Informed consent for all participants was obtained according to the Declaration of Helsinki (1991), and protocols and procedures were approved by the relevant local ethical committee at each site. The cohorts as mentioned above were genetically analysed to identify known or novel non-synonymous variants in exon 2 of the *TREM2* gene, previously linked to pathogenic risk or predicted to be detrimental. Of the 48 participants identified with a *TREM2* pathogenic variant (TREM2^var^, Table 1), 10 were controls, 10 had MCI and 28 had a dementia diagnosis (AD). Similar age-matched non-carrier control (*n* = 10), MCI (*n* = 8) and AD (*n* = 31) samples were also included.

**Table 1:** Demographic and clinical characteristics of *TREM2* rare variant carriers and non-carriers

	*TREM2* non-carriers (*n* = 49)	*TREM2* rare variant carriers (*n* = 48)	*TREM2* rare variant carriers			
			p.R47H (*n* = 26)	p.T96K (*n* = 8)	p.D87N (*n* = 5)	Others (*n* = 9)
Age, years (SD)	76.1 (6.7)	75.2 (7.3)	73.9 (8.4)	76.6 (4.8)	72.4 (6.8)	76.9 (4.1)
Female, *n* (%)	27/49 (55.1)	26/48 (54.2)	12 (46.2)	5 (55.5)	3 (60)	6 (75)
*APOE* ɛ4 carriers, *n* (%)	24 (68.6)^a^	21 (59.7)^b^	11	3	1	6
MMSE, *n* (SD)	23.3 (5.0)	23.1 (6.4)	21 (7.5)	25 (5.0)	28.8 (4.5)	24.7 (2.3)
Diagnosis, *n* (%)	AD, 31/49 (63.3); MCI, 8/49 (16.3); Ctrl, 10/49 (20.4)	AD, 28/48 (58.4); MCI, 10/48 (20.8); Ctrl, 10/48 (20.8)	18/26 (69); 4/26 (15.5); 4/26 (15.5)	2/9 (25.0); 3/9 (37.5); 3/9 (37.5)	1/5 (16.7); 2/5 (33.3); 2/5 (50.0)	7/9 (87.5); 1/9 (12.5); 0/9
sTREM2, ng/L (SD)	8750 (5265)	7346 (5526)	7294 (6791)	8761.8 (4840)	6431 (4107)	7009 (1889)
NFL, ng/L (SD)	26.1 (17.1)	24.6 (19.1)	25.7 (23.8)	25.2 (17.8)	23.1 (7.7)	21.3 (7.6)

a = 14 individuals with missing *APOE* status

b = 13 individuals with missing *APOE* status

### Plasma measures of sTREM2 and NFL

Plasma sTREM2 was measured using an in-house electrochemoluminescent assay on the MesoScale Discovery SECTOR imager 6000 (MesoScale Discovery (MSD), Maryland, USA) using a method adapted from Kleinberger et al. [29]. The capture antibody was the biotinylated polyclonal goat anti-human TREM2 (0.25 μg/mL, R&D Systems, Minneapolis, USA), and the detector antibody was monoclonal mouse anti-human TREM2 (1 μg/mL, Santa Cruz Biotechnology, Texas, USA). A standard curve for calculations of unknowns was constructed using recombinant human TREM2 (4000–62.5 pg/mL, Sino Biological, Bejiing, China), and plasma samples were diluted 1:4 before being assayed. For a more comprehensive description of the method, please see [29]. For NFL, the commercially available NF-light assay on an HD-1 Simoa instrument (Quanterix, Lexington, MA, USA) was utilized. All biochemical analyses were performed at the Institute of Neurology at University College London (UCL).

### Sample size and power calculation

In CSF, the effect size of sTREM2 between AD and control ranges between 1.077 and 1.539 (mean 1.272; source: Alzbiomarker, Alzforum). In applying a type error I (α) of 0.05, we reach a power (1-β) of 0.99 in our sample size of 97 participants (G*Power). However, the effect size of plasma sTREM2 is likely to be considerably lower than that of CSF sTREM2. Therefore, we examined the achieved power as a function of effect size (Cohen’s *d*), with a type error I (α) of 0.05 assuming 97 participants. In a medium effect size (Cohen’s *d* = 0.5), a power of 0.68 is achieved, whereas a large effect size (Cohen’s *d* = 0.8) computes a power of 0.97. Thus, we can reasonably state that there is a low probability of error type II in our results if the effect size was large.

### Statistical analysis

Data normality was determined by the D’Agostino-Pearson test, and statistical evaluation was performed on log_10_-transformed data. After transformation, the data followed a normal distribution. All data analysis reported has been performed on log_10_-transformed sTREM2 and NFL, but the untransformed values are shown in descriptive tables and figures. To study the association of plasma measures with demographic data, a Pearson product-moment correlation was utilized for age and Mini-Mental State Examination (MMSE) whereas *t* test (sTREM2) or a one-way analysis of covariance (ANCOVA, NFL) for gender and APOE status. Only age was a significant predictor of plasma NFL; the subsequent analyses were therefore conducted including age as a confounder. A *t* test or ANCOVA were conducted to determine clinical group differences between blood biomarkers. ANCOVA analyses were followed by a Bonferroni-corrected post hoc pairwise comparison where appropriate. A partial correlation, adjusted by age, tested the association between plasma sTREM2 and plasma NFL. Statistical analysis was performed using IBM SPSS Statistics, version 25 (Armonk, NY, USA).

## Results

Forty-eight confirmed *TREM2* rare variant carriers and 49 non-carriers were included in the study. The *TREM2* rare variant carrier group comprised of 10 different variants: p.Q33X, p.D39N, p.R47H, p.P59L, p.R62H, p.D87N, p.T96K, p.Q691H, p.H703Y and p.L868R. The demographical characteristics of the cohort are described in Table 1. There were no differences in age between *TREM2* rare variant carriers (M = 75.2, SD = 7.3) and non-carriers (M = 76.1, SD = 6.7). The inclusion of gender was very similar across groups (carriers, 26/48 female [54.2%]; non-carriers, 27/49 female [55.1%]). Finally, there was no statistically significant difference in MMSE (carriers, M = 23.1, SD = 6.4; non-carriers, M = 23.3, SD = 5.0) or clinical diagnosis between the two groups (Table 1).

**Fig. 1:**
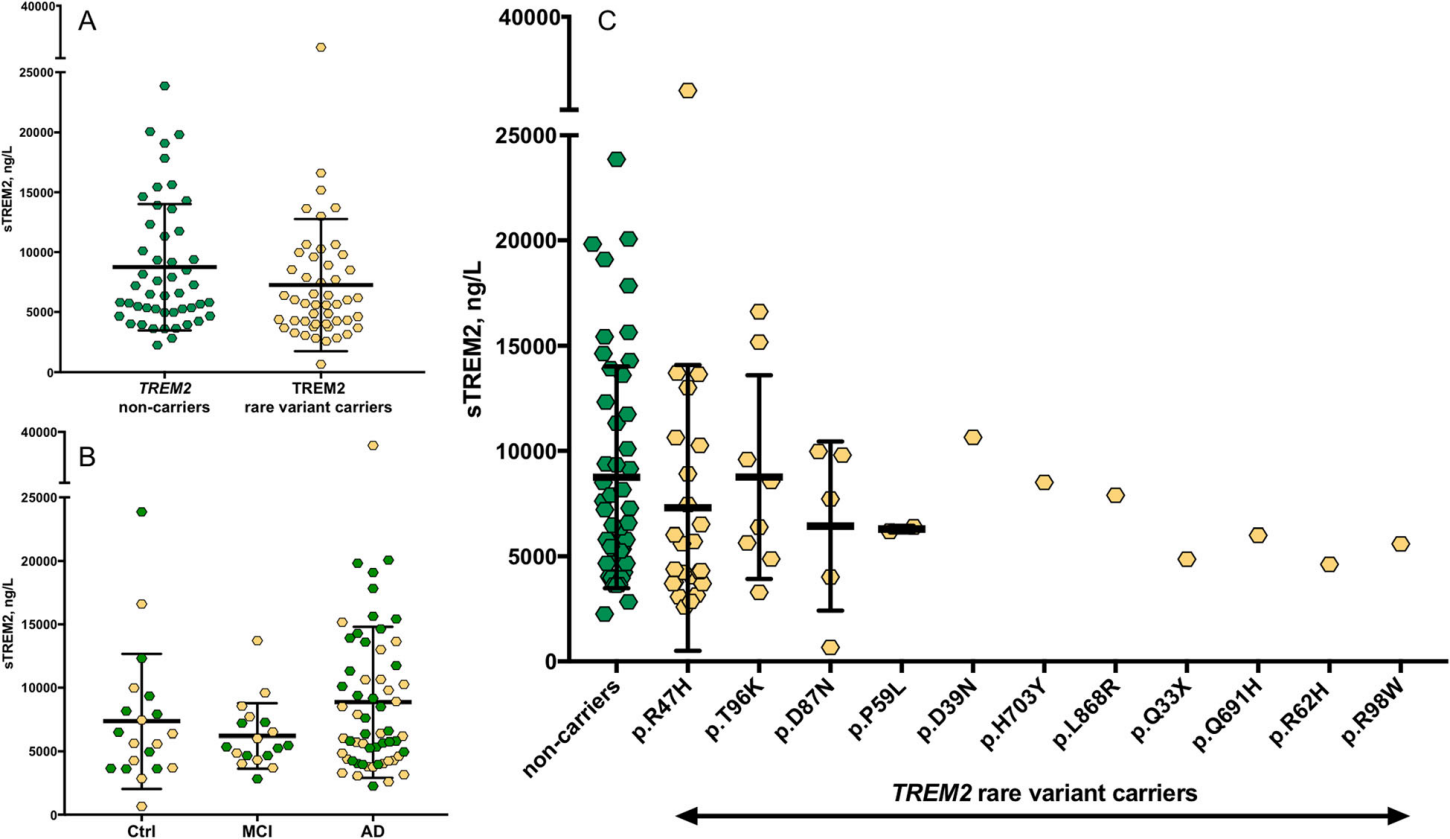
The concentrations of plasma sTREM2. No change in the levels of sTREM2 between *TREM2* rare variant carriers and non-carriers (**a**, **b**). A non-significant increase in sTREM2 was observed in AD patients compared to MCI and aged-matched controls (**c**)

In the whole cohort, plasma sTREM2 was not associated with age (*r* = 0.060; *P* = 0.562), gender (*P* = 0.083), *APOE* ε4 status (*P* = 0.237) or MMSE (*r* = − 0.018; *P* = 0.858). There were no differences in the levels of plasma sTREM2 between *TREM2* rare variant carriers (M = 7346 ng/L, SD = 5526 ng/L) and non-carriers (M = 8750 ng/L, SD = 5265 ng/L; *t*(95) = 1.696, *P* = 0.093; Fig. 1a). There were no significant differences in plasma sTREM2 between carriers of different *TREM2* rare variants (*F*(3, 84) = 1.68, *P* = 0.177, Fig. 1b). Note, we only included *TREM2* rare variants with > 2 individuals per group in this analysis. Adding clinical diagnosis as covariate did not change the result (*P* = 0.171). Next, we tested whether plasma sTREM2 levels differ between clinical diagnoses, regardless of the *TREM2* rare variant status, between AD (M = 8859 ng/L, SD = 5951 ng/L), MCI (M = 6204 ng/L, SD = 2572 ng/L) and controls (M = 7352 ng/L, SD = 5318 ng/L). Plasma sTREM2 levels were not different between these groups (*F*(2, 94) = 1.84, *P* = 0.164, Fig. 1c). Adjusting for the effect of age and gender did not change the result of sTREM2 in plasma.

**Fig. 2:**
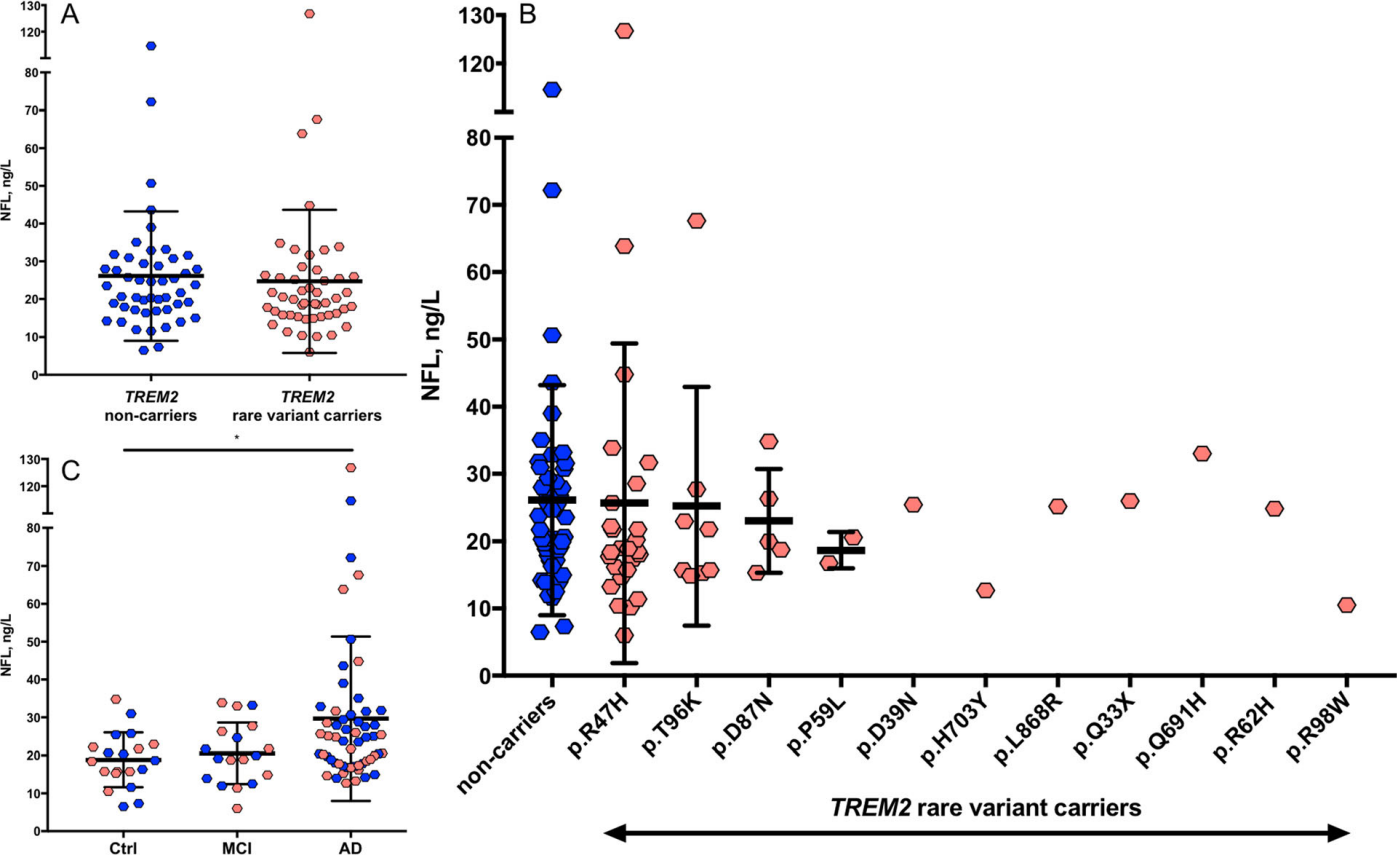
The concentrations of plasma NFL. No change in the concentrations of NFL between *TREM2* rare variants and non-carriers (**a**, **b**). A significant increase in NFL was observed in AD patients compared to MCI and aged-matched controls which was not influenced by *TREM2* mutation status (**c**)

As expected, plasma NFL concentrations were significantly associated with age (*r* = 0.202, *P* = 0.047). After accounting for the effect of age, plasma NFL levels were found to be associated with MMSE (*r* = − 0.353, *P* = 0.0004) and tended to be higher in females (M = 25.6, SD = 13.1 ng/L) compared to males (M = 25.1, SD = 22.8 ng/L; *P* = 0.079). Levels were not affected by *APOE* ε4 status (*P* = 0.899). There were no differences in plasma NFL between *TREM2* rare variant carriers (M = 24.6 ng/L, SD = 19.1 ng/L) and non-carriers (M = 26.1 ng/L, SD = 17.1 ng/L) (*F*(1, 94) = 0.505, *P* = 0.479, Fig. 2a), after adjusting for the effect of age. Similar to plasma sTREM2, there were no differences in plasma NFL when comparing the different *TREM2* rare variants (*F*(3, 83) = 0.113, *P* = 0.952, Fig. 2b). This remained true when correcting for the effect of the clinical diagnosis (*P* = 0.633). As expected, differences in plasma NFL were observed between the three clinical groups (*F*(2, 93) = 4.89, *P* = 0.010). The Bonferroni-corrected post hoc pairwise comparison demonstrated that the AD group (M = 29.0 ng/L, SD = 21.6 ng/L) had significantly higher levels of NFL compared to controls (M = 18.8 ng/L, SD = 7.2 ng/L; *P* = 0.025) but not to MCI (M = 20.5 ng/L, SD = 8.1 ng/L; *P* = 0.096, Fig. 2c). A further adjustment for the effect of gender did not change these NFL findings.

In the whole cohort, when correcting for age, a significant positive correlation between plasma sTREM2 and plasma NFL was observed (*r* = 0.245, *P* = 0.016). However, this correlation is driven by the symptomatic individuals (MCI and AD) as no significant association of plasma sTREM2 and plasma NFL was observed in the control group (*r* = 0.250, *P* = 0.302) but a tendency in the symptomatic group (*r* = 0.223, *P* = 0.053).

## Discussion

This is the first study to have comprehensively investigated if the levels of plasma sTREM2 and a neurodegenerative marker, plasma NFL, differ between *TREM2* rare variant carriers and non-carriers. Our main finding demonstrates that there are no significant differences in plasma sTREM2 and NFL between these groups. Furthermore, we also show that plasma sTREM2 does not differ between controls, MCI and AD.

While sTREM2 has been extensively studied in CSF, only a few studies have reported sTREM2 in blood. Piccio and co-workers [28] demonstrated that serum sTREM2 levels did not differ between multiple sclerosis, other inflammatory neurologic diseases and non-inflammatory controls. Kleinberger et al. [29] found no difference in plasma sTREM2 between healthy controls, AD and FTD. The lack of separation between AD and controls was then independently replicated [32]. In a different approach, Ohara et al. showed that increased serum sTREM2 is associated with increased risk to develop dementia in Japanese population [42]. Herein, we also demonstrate that plasma sTREM2 is not different between *TREM2* rare variant carriers and non-carriers, nor with respect to clinical diagnosis. Therefore, although readily detectable, sTREM2 in blood is not useful to discriminate those people with a clinical diagnostic group or those with a *TREM2* variant associated with AD. It should be noted that sTREM2 in blood most likely has a peripheral rather than a central nervous system (CNS) origin from microglia. This is in agreement with the ubiquitous expression pattern of TREM2 at both mRNA and protein levels in Human Protein Atlas (https://www.proteinatlas.org/ENSG00000095970-TREM2/tissue). TREM2 is highly expressed in cells from a myeloid lineage, such as monocytes, macrophages, Kupffer cells or osteoclasts [43–46].

Previously, it was shown that both plasma and CSF sTREM2 were useful to detect those with homozygous *TREM2* mutations (e.g. p.T66 M, p.W198X, p.Q33X and p.Y38C) that lead to Nasu-Hakola disease or an FTD-like syndrome [47, 48]. In these diseases, sTREM2 in blood or CSF is almost absent, which is in line with the impaired cell surface transport and shedding that occur with these mutations [29, 49]. In contrast, heterozygous *TREM2* rare variant carriers have a less obvious and inconsistent pattern of CSF sTREM2. While the *TREM2* p.R47H rare variant is associated with increased CSF sTREM2, other *TREM2* variants (e.g. p.L211P; T96K/L211P/W191X) have been reported to be associated with decreased or unchanged levels of CSF sTREM2 (e.g. R62H) [32, 33] (Deming et al. Sci Trans Med 2019 *in press*). Our findings could be explained by how these rare variants differently affect the processing and shedding of TREM2. For example, individual variants are believed to impact TREM2 production (Q33X), expression or turnover at the cell surface (R47H) [29, 50]; α-secretase cleavage of the extracellular soluble ectodomain (H157Y) [27, 51]; and/or ligand binding (R47H, R62H, T96K, H157Y) [52–54]. Surprisingly, we did not find such differences in plasma, suggesting a different regulation of TREM2 in the periphery and the CNS. We also investigated plasma NFL, but here, we also found no differences between *TREM2* rare variant carriers and non-carriers, even when introducing clinical diagnosis as a co-variable, suggesting that the neuronal injury is no different in AD irrespective of whether someone has a *TREM2* rare variant. These findings are agreement with clinical data that find AD cases with a *TREM2* p.R47H rare variant are clinically indistinguishable from other AD, albeit those with a *TREM2* rare variant generally have an earlier age of onset [55]. Finally, the finding that sTREM2 is associated with NFL in AD is consistent with the association of CSF sTREM2 with T-tau, another marker of neurodegeneration, and suggests that inflammatory response might be coupled to neurodegeneration [56].

This study has some limitations. Despite our study recruiting the highest number of *TREM2* rare variant carriers to date, some individual variants were represented by only very small numbers, which precluded a comparison between them. Additionally, these samples did not have the core AD CSF biomarkers to confirm diagnosis, disease stage or link analyses individually to tau or amyloid burden, and hence, we were limited to using clinical diagnosis. The main strengths are the fact that we used very reliable and well-established assays for both sTREM2 and NFL. The study was designed in such a way that an important total number of *TREM2* rare variants were carefully matched to a control group in terms of age, gender and clinical diagnosis.

## Conclusion

This study, for the first time, demonstrates that the levels of sTREM2 and NFL in plasma do not differ between *TREM2* rare variant carriers and non-carriers. Furthermore, we confirm previous reports that sTREM2 is not changed in AD or MCI compared with aged-matched controls. Therefore, we conclude that although plasma sTREM2 may be useful to detect *TREM2* homozygous mutations, plasma sTREM2 is not a reliable biomarker to detect *TREM2* rare variant status nor suspected AD.

## Data Availability

The datasets used and/or analysed during the current study are available from the corresponding author on reasonable request.

## Abbreviations

AD: Alzheimer’s disease
ALS: Amyotrophic lateral sclerosis
ANCOVA: Analysis of covariance
CNS: Central nervous system
CSF: Cerebrospinal fluid
FTD: Frontotemporal dementia
MCI: Mild cognitive impairment
MMSE: Mini-Mental State Examination
MSD: MesoScale Discovery
NFL: Neurofilament light chain
PD: Parkinson’s disease
P-tau: Phosphorylated tau
Simoa: Single molecule array
TREM2: Triggering receptor expressed on myeloid cells 2
T-tau: Total tau
